# The Modality-Specific Learning Style Hypothesis: A Mini-Review

**DOI:** 10.3389/fpsyg.2018.01538

**Published:** 2018-08-21

**Authors:** Karoline Aslaksen, Håvard Lorås

**Affiliations:** ^1^Department of Psychology, Faculty of Social and Educational Sciences, Norwegian University of Science and Technology, Trondheim, Norway; ^2^Department of Neuromedicine and Movement Science, Faculty of Medicine and Health Sciences, Norwegian University of Science and Technology, Trondheim, Norway

**Keywords:** modality-specific, instruction, teaching, learning styles, meshing hypothesis, neuromyth

## Abstract

The impact on learning outcome of tailoring instruction and teaching toward modality-specific learning style preferences has been researched and debated for decades. Several topical reviews have concluded that there is no evidence to support the meshing hypothesis and that it represents a persistent neuromyth in education. The concept, however, is still utilized in educational practice and favored by many academics. This mini-review presents literature, which has applied explicit and rigorous methodological criteria, in relation to the meshing hypothesis. In order to demonstrate evidence for the meshing hypothesis, studies had to screen participants for their preferred learning style, assign participants to matched or non-matched conditions, and then provide the same test to assess learning for all participants, as well as presenting statistical crossover-interaction effects. Across studies that have applied these methodological criteria, the overall effect sizes were very low and non-significant, indicating that there is still no replicable statistical evidence for enhanced learning outcome by aligning instruction to modality-specific learning styles.

## Introduction

The concept of matching instructional strategies to an individual’s learning style in order to enhance learning outcome and achieve better academic success is a well-known concept among educators and the general population (Pashler et al., 2008; Dekker et al., 2012; Howard-Jones, 2014). Learning styles are considered to have an impact in any learning situation regardless of content and this *“refers to the concept that individuals differ in regard to what mode of instruction or study is most effective to them"* (Pashler et al., 2008). The term learning styles first appeared in the literature many decades ago (e.g., Thelen, 1954) and has been the focus of extensive research for the past three decades, especially in Western Europe and the United States (Coffield et al., 2004).

Amongst a plethora of concepts and perspectives on learning styles (see Coffield et al., 2004, for a tour de force on learning style concepts), one of the most cited and well-known learning style perspectives concerns modality-specific preferences (Coffield et al., 2004; Howard-Jones, 2014; Cuevas, 2015). The overall prediction is that if individuals are given instruction in their preferred modality (visual, auditory, or kinesthetic), they will experience enhanced learning outcomes. This has been termed the *meshing hypothesis* (Pashler et al., 2008). A related perspective that offers basically the same prediction states that people who are “verbalizers” will perform better if they are given verbal instructions and that “visualizers” will perform better if instructions are presented visually (Massa and Mayer, 2006; Kollöffel, 2012). In either perspective, the instructional method should *mesh* with the preferred modality-specific learning style. The learning style concept, in general, and the meshing hypothesis, in particular, have been subjects of tremendous scrutiny in the recent years that continues to the present. Several independent authors have advanced the view that the latter represents a *neuromyth*, a term applied to educational applications argued to be based upon popular perspectives of brain functioning (Geake, 2008; Riener and Willingham, 2010; Dekker et al., 2012; Howard-Jones, 2014; Newton, 2015; Newton and Miah, 2017). Typically, the evidence for neuromyths does not correspond to the findings of studies from cognitive psychology and the neurosciences, and sometimes the scientific evidence contradicts the brain-based claims (Geake, 2008). In terms of the meshing hypothesis, the implicit assumption is that the learning material delivered via one sensory modality (i.e., visual, auditory, or kinesthetic) is processed in the brain independently from material delivered via other sensory modalities. However, substantial scientific evidence shows support for cross-modal processing and interconnectivity that contradicts the meshing perspective and demonstrates that input modalities in the brain are always interlinked (Calvert et al., 2000).

The overall claim for improving learning by matching the mode of instruction to modality-specific learning preferences independent of both ability and content (Riener and Willingham, 2010), as reflected by the meshing hypothesis, has also been scrutinized in several literature reviews. At first sight, modality-specific instruction appears to be supported by a large body of empirical literature (Rohrer and Pashler, 2012). However, upon closer inspection, few of these studies have been found to have an appropriate research design (Pashler et al., 2008). First, subjects need to be divided according to their preferred learning style, e.g., visual or auditory learners, based upon some sort of learning style assessment. Second, studies with an appropriate design must then randomize subjects (regardless of their assessed learning style) to receive either instruction tailored to their style or instruction tailored for other learning styles. This asserts that some subjects were presented with the “correct” kind of instruction (i.e., aligned with their preferred learning modality) and some with the “incorrect” instruction. Finally, all participants must be administered the same test to assess learning, and the results would support the efficacy of the practice of aligning instruction with modality-specific learning style if, and only if, the test scores reveal that, e.g., visual learners do better if instruction is presented visually rather than auditorily, and likewise, auditory learners do better if instruction is presented auditorily rather than visually (crossover-interaction effects; Pashler et al., 2008). In previous reviews, it has been stated repeatedly that there is a lack of studies that employ this rigorous design and that the few available at the time have, overall, generated no evidence to support the meshing hypothesis (Coffield et al., 2004; Kozhevnikov, 2007; Pashler et al., 2008; Willingham et al., 2015).

The disappointing outcome of all these empirical and theoretical endeavors and efforts is that the modality-specific learning style concept is, as stated by Newton (2015), *thriving* across all levels of education. This is reflected in the findings of 89% of research papers published from 2013 to 2015 and located in ERIC and PubMed databases support the application of learning styles to instructional methodology (Newton, 2015). Furthermore, a survey by Dekker et al. (2012) showed that 93% of United Kingdom primary and secondary school teachers assumed that *“individuals learn better when they receive information in their preferred learning style.”* Later studies have revealed similar findings in other countries, K-12 teachers responding positively to statements favoring modality-specific learning styles (Howard-Jones, 2014; Gleichgerrcht et al., 2015; Ferrero et al., 2016). In addition, when faculty working in higher education in the United States were given the following question: *Does teaching to a student’s learning style enhance learning?*, approximately two-thirds answered in the affirmative (Dandy and Bendersky, 2014). At the institutional level, Meyer and Murrell (2014) found that, across 39 educational institutions in the United States, more than 70% taught “learning style theory” as a topic in teacher education.

A recent study showed a downward trend for the general belief in learning styles among academics working in higher education in the United Kingdom (*n* = 114), although 58% still report believing in the concept and about a third report using learning styles actively in their work (Newton and Miah, 2017). Thus, there appears to be widespread acceptance among educators, students, and academics globally and across all levels of education that the concept of learning styles is an established, textbook principle. Indeed, texts used in teacher education courses present learning style theory as a way to differentiate instruction for students (Cuevas, 2015).

The presented considerations demonstrate that there exists a substantial continuum of perspectives on the application of modality-specific learning styles, ranging from viewing the concept as a neuromyth that should be abandoned in pedagogical practice to those who speak in favor of the concept and might use it as part of their routine practices. The principal aim of this mini-review is to provide a contribution toward narrowing this gap in perspectives by providing an updated overview of the available empirical studies that have applied rigorous methodological criteria as outlined by Pashler et al. (2008). To the best of the authors’ knowledge, although previous reviews touching upon modality-specific learning styles have been both thorough and in-depth, they have been mostly narrative and have not been accompanied by a focus on specific effect sizes. This latter approach can be important for disentangling divergences in results, as there might be disagreements among studies. Pooling methodological and conceptually similar studies that all involve a certain degree of error allows for deriving an estimate of overall effect size that considers contrasting results from different studies. Such an update seems timely, given that several studies with methodological rigor have been published since the previous reviews.

## Scope of the Mini-Review: Selection Criteria for Reporting of Evidence

The aim of this mini-review was to present literature in relation to the meshing hypothesis. Consequently, the authors independently performed database searches in EBSCO (including ERIC, Academic Search Complete, Psychology and Behavioral Sciences Collection) and Ovid (including Medline, EMBASE, and PsychINFO) using combinations of the terms *learning styles^∗^*, *visual^∗^*, and *auditory^∗^*. The searches were conducted up to January 2018. The reference lists from previous reviews were also examined, as well as citation-based searches in Google Scholar. A total of 1215 records were initially scanned, and 10 studies (Constantinidou and Baker, 2002; Massa and Mayer, 2006; Kassaian, 2007; Korenman and Peynircioglu, 2007; Slack and Norwich, 2007; Tight, 2010; Kollöffel, 2012; Hansen and Cottrell, 2013; Rogowsky et al., 2015; Papanagnou et al., 2016) were found that had applied the appropriate methodology according to the criteria by Pashler et al. (2008).

## Tailoring Instruction for Modality-Specific Preferences: No Statistical Evidence for the Meshing Hypothesis

Statistical evidence for the meshing hypothesis could potentially be found in crossover-interaction effects, i.e., visual learners demonstrate improved learning if instruction is visual rather than auditory, and likewise, auditory learners show improvements if instruction is auditory rather than visual. The 10 publications amounted to 13 experiments, from which it was possible to extract means (SD) for computation of effect sizes (Hedges’ *g*) for 11 of them. Altogether, 22 effect sizes from post-test data representing the differences in scores between the matched groups and the mismatched groups were analyzed by a random effects model. This resulted in a small and non-significant effect size for visual matching (*g* = -0.09, 95% CI [-0.74–0.58], *p* = 0.80, *n* = 484) as well as for auditory matching (*g* = -0.27, 95% CI [-0.87–0.32], *p* = 0.37, *n* = 356). In the paper by Constantinidou and Baker (2002), the authors did not report data that allow for the computation of Hedges’ *g*. The authors did state, however, that no significant correlation between learning style and experimental task performance was found. Similarly, Papanagnou et al. (2016) reported only mean values for matched/non-matched learning outcomes and stated that both matched and non-matched groups achieved similar learning outcomes. Based on these data, it thus appears that there is no replicable evidence for a statistical crossover-interaction effect where participants systematically show higher learning outcomes when they are in a condition in which their preferred learning style modality matches the instructional mode and a lower learning outcome when there is a mismatch.

The overall (non-significant) effect sizes obtained across studies appears to be, by any standard, too small to be interpreted as signifying any modality-matching effect on learning outcomes. Although the interpretation of effect sizes is not a straightforward scientific endeavor (Cohen, 1992), the effect size cut-offs indicating a practically relevant effect provided in the literature represent a much more substantial magnitude. For example, Ferguson (2009) recommended that a minimum effect size representing a “practically” significant effect amounts to *g* ≥ 0.41, and Hattie (2009) has advanced the view that effect sizes ≥0.40 represent a “hinge-point” at which deliberate interventions provide relevant outcomes for teaching and learning. Adding to the overall interpretation of the effect sizes obtained in the current meta-analysis, the 95% confidence intervals demonstrate crossings of zero both for the overall effect size and in data from some individual studies. This latter finding is a strong indicator that the null hypothesis (no effect of modality matching) should not be rejected (Wilkinson et al., 1999).

An often-stated problem in the learning style literature is the plethora of inventories designed and applied for both research and commercial purposes (Coffield et al., 2004; Peterson et al., 2009; Scott, 2010; Armstrong et al., 2012). At first sight, this might appear as a methodological challenge toward the pooling of results across studies. In particular, the VAKT classification vs. the verbalizer–visualizer dimension have previously been advocated as different and non-comparable approaches toward learning styles; e.g., it has been claimed that the verbalizer–visualizer dimension should be defined as a cognitive style and not included among the “family” of learning styles (Massa and Mayer, 2006; Kollöffel, 2012). However, the latter perspective involves modality-specific content. Written material is considered proper instruction for verbalizers, as it is processed as spoken words, and therefore, a verbalizer can be considered synonymous to an auditory learner (Felder and Silverman, 1988). Based on these contentions, there are strong theoretical arguments for a comparison of studies applying inventories based upon either perspective. Furthermore, rarely is any theoretical or methodological argument for the inclusion of a specific inventory in studies given, and in addition, some authors advance the view that one should apply the inventories that are most used (or most popular) in order to generate comparable results (Hansen and Cottrell, 2013).

There still appear to be relatively few studies adhering strictly to the methodological criteria outlined by Pashler et al. (2008). In particular, the participants’ learning styles are not necessarily established before they are separated into groups (e.g., Korenman and Peynircioglu, 2007), and participants can be randomly assigned to either *one* (Massa and Mayer, 2006; Rogowsky et al., 2015) or *all* conditions (e.g., Kassaian, 2007). The only study located through the systematic literature search across six different databases and the screening of more than a 1000 records that was totally aligned with Pashler’s criteria was Rogowsky et al. (2015). These authors report no statistically significant relationship or crossover-interaction effect between modality-specific learning styles and modes of instruction. Here, the authors assessed the participants’ learning styles and randomly assigned participants to either listening to a digital audiobook or reading an e-text, and all participants completed the same achievement test. Interestingly, the effect sizes from this latter study (visual-matching: *g* = -0.11, auditory-matching: g = -0.256) were similar to the overall effect size across studies.

The experimental tasks applied in studies varied considerably. The pooling of such various approaches can be justified by the modality-specific learning style theory. Here, the basic contention is that modality matching introduces more efficient learning *irrespective* of content and contexts. Indeed, the concept of a modality-specific learning style has been featured in the literature as a hardwired and more or less inherited preference in the cognitive system that should be taken into consideration in any learning situation (Coffield et al., 2004). One methodological concern, however, arises when examining learning tasks more closely. It appears that some tasks have a “built-in” stronger visual or auditory component, which could potentially introduce an additive bias in favor of both a particular instructional mode and a learning style (Fiorina et al., 2007; Hansen and Cottrell, 2013; Willingham et al., 2015). Although this could potentially lead to inflated effect sizes, the overall pattern of results across studies suggested no statistical effect of modality matching.

As stated in the introduction, the modality-specific learning style hypothesis is still a favored concept amongst the general public, educators, and in the research literature (Pashler et al., 2008; Dekker et al., 2012; Howard-Jones, 2014). In previous reviews, it has been systematically addressed that there is, in general, no evidence to support the application of the learning style concept (Coffield et al., 2004; Desmedt and Valcke, 2004; Kozhevnikov, 2007; Pashler et al., 2008; Peterson et al., 2009; Cuevas, 2015; Willingham et al., 2015). The present study responds to a call from the much-cited review (>1,500 citations in Google Scholar) of Pashler et al. (2008), who stated that, in order for the learning styles hypothesis to be supported, several well-designed studies would have to test, amongst other elements, the modality-matching hypothesis and show significant interaction effects. Although the total number of studies (*n* = 10) with appropriate methodology is not large at this time, the pattern of results clearly leans toward showing that tailoring instruction/teaching toward preferred modality-specific learning styles has no effect on learning outcome/rate.

## Concluding Remarks

This mini-review has demonstrated that, across studies that have applied equivalent quantitative empirical research designs, no overall improvement in learning outcome when applying modality-specific matching of instruction was found. This conclusion of the presented meta-analysis of an element of the modality-specific learning style literature appears to add to further evidence-based refutations of the meshing hypothesis. Interestingly, some early meta-analysis on other elements of learning styles presented similar conclusions (Tamir, 1985; Kavale and Forness, 1987). This appears in contrast to the recent literature review by Newton (2015), in which it was demonstrated that a considerable percent (89%) of published studies in the period from 2013 to 2015 was positive toward learning styles. It thus appears important to continue to critically scrutinize different aspects of the learning style literature and to conduct pattern-type explanations (Derry, 1999) involving conceptual syntheses of insights emerging from diverse disciplines. For example, connections have been found between visual-spatial strengths and superior abilities in other cognitive domains (O’Boyle et al., 2005; Root-Bernstein et al., 2008). This latter work is not typically connected with modality-specific learning styles in the academic literature and highlights the need for further work on the credibility of the meshing hypothesis in order to prevent potential misuse of what might appear to be a persistent neuromyth.

## Author Contributions

All authors listed have made a substantial, direct and intellectual contribution to the work, and approved it for publication.

## Conflict of Interest Statement

The authors declare that the research was conducted in the absence of any commercial or financial relationships that could be construed as a potential conflict of interest.
